# The Mystery of Dreams

**Published:** 1875-09

**Authors:** 


### THE MYSTERY OF DREAMS.

We speak of things as wonderful after the manner they impress us; so that which is a source of astonishment to one person, may produce no such emotion in another. Of course, in an absolute sense, one phenomenon of thought is as marvellous as another, for how we think at all is an inscrutable mystery. Still, in dreams, there is so much that apparently differs from the mental operations of our waking hours, that the experience is often more remarkable and impressive.

What dreams are, all know by their own experience. By some a good deal of importance is attached to them. In the oldest books extant they are mentioned, and a supernatural origin generally ascribed to them. The Bible clearly shows this as well as the poems of Homer. In the ancient Oriental courts of Babylon and Egypt it was customary for monarchs to have a class of persons about them whose business it was to interpret dreams, and this was an important office of state. The classics abound in evidences of the great, widespread faith in their spiritual nature. Grave philosophers have written treatises on their interpretation as they did on astrology. A common way of consulting the Greek and Roman oracles was for the inquirer to sleep a night in the temple, after the due performance of religious rites, when his questions were supposed to be answered in dreams. That dreams should have a peculiar significance attached to them by the ignorant and unlettered is not to be wondered at when we consider some of their extraordinary phenomena and the proneness of the mind to be affected by what is marvellous. They often come without any logical connection with what is remembered. They produce impressions frequently deeper than any written or spoken thought. They are sometimes characterized by a clearness of vision and a definiteness of aim that give them a peculiar emphasis and influence. Often, we know, they are a vague jumble of notions, or an incoherent series of images, or a terrible sense of the awful and hideous, or a ludicrous picture of improbable situations and circumstances, or a fantastic and vanishing display, in which we are alternately pleased and distressed. Then again they are signalized by the very highest operations of mind. For in dreams persons have found the clue to hidden paths of discovery, have solved the deepest problems, have composed admirable poems and music, have had what they accepted as providential warnings and prophetic helps to the life before them. All who have any knowledge of the subject are aware that the bodily condition and the previous mental state have a great deal to do with these experiences. A full meal before sleeping, a peculiar train of thought during the day, unusual joy or sorrow, any extraordinary sensations, any strong excitement or solicitude, will be likely to shape and modify one's dreams. All of us have been the subject of states in sleep that surpass even the wildest imaginings of the day. We have seemed to struggle with the most terrible difficulties, to be plunged to the darkest abysses, and to be lifted to supreme heights of blessedness. We have swam through atmospheres of light and joy, have walked with the absent amid distant yet familiar scenes, have rejoined the loved in the everlasting meadows of heaven, have been in the midst of demons and of angels, have been conquered in awful conflicts, and have come off victors ourselves, have died, have risen to peace, and have had life set to all possible activities and employments. Indeed, there is nothing too fantastic or improbable in the range of our lives that we have not experienced or had glimpses of in dreams.

The dream, while it continues, is unquestionably a real experience. If one should continue to dream, the state, to all intents, would be as actual as any other. Now we know that the consciousness to which we give the name of dream is occasioned and modified largely by the peculiarity of the sleeper's sensations at the time. Dr. Gregory relates that, having occasion to apply a bottle of hot water to his feet at bedtime, he dreamed that he was treading the hot soil of Mt. Etna, and when Dr. Reid once applied a blister to his head he dreamed that he was scalped by Indians. These illustrations might be indefinitely extended, but almost every one can corroborate the fact affirmed by experience to some significant experience of his own, of a similar kind. By affecting, therefore, the organs of sense, or any of the vitalities of our being so that the mind is reached, there may be produced impressions in sleep of the most significant character. Now it is with joy and suffering that we are most concerned, and which in some way comprise what is most important to us in life. But dreams show a wonderful scope—a prodigious capacity of our nature for such experiences. There have been opened to the sleeper elysiums of transcendent happiness. He has been touched by harmonies more exquisite, been thrilled with utterances more sublime, been tranced in ecstasies more supernal, been dazzled with glories more sweet and ravishing than any that ever signalized his waking state. In fact, he has been more profoundly convinced of the possibility of a rarer and deeper enjoyment than any yet realized, by some wonderful dream, than by any other influence. He may not be able to define the quality of the good that he had a glimpse of in sleep, nor give a name to the disclosure, nor impart to a listener the consciousness that he had of light-life above, but the glorious impression was strongly made. It told what was possible within the limits of our existence under other and suitable conditions. And so with the opposite impression of suffering. Who has not at some time while in slumber, had an indescribable sense of horror most intolerable and overwhelming—something that has no apparent analogy to any ordinary pain or dread, but is of a kind by itself in the suggestions of supernatural agony. De Quincy describes some of his hallucinations under the influence of opium, and among other things says: "I fled from the wrath of Brahma through all the forests of Asia. Vishnu hated me, Seeva lay in wait for me. I came suddenly upon Isis and Osiris. I had done a deed, they said, at which the ibis and the crocodile trembled. I was burned for a thousand years in stone coffins with mummies and sphinxes, in narrow chambers at the heart of eternal pyramids. I was kissed by cancerous kisses of crocodiles, and lay confounded with unutterable slimy things among reeds and Nilotic mud."

Sometimes the sufferer is clearly conscious of the scenery, personages, and events, through whose terrible panorama comes his sense of woe—everything is sharply defined in the vision of the soul. But again he is the subject of more sensations of dread that pierce even to the marrow of his bones—great black, undefined, gigantic loads of misery seem pressing him down—strange, subtle, brutal, demoniac influences seem to have him in their control; he is stifled by a breath more leprous and infernal than any conception that he imagines of disgust. The very horror of the experience rouses him, and he is never so thankful as when he knows it is all a dream. And yet there is most solemn meaning in all this, whether it be of pain or pleasure to the sleeper, who perchance soon forgets the impression that for a while was so powerful and thrilling. We are forced to allow, by considerations like these, that our capacity for enjoyment or suffering is tremendous, and far beyond anything that may be inferred from the usual phenomena of our experience. We see that there are possibilities that we have not yet fathomed in our actual being but which may be as natural as any of the ordinary operations of our lives.

But this view of what the human soul has capacity for, as indicated by dreams, has powerful emphasis in another phenomenon of their appearance, the astonishing rapidity of the operation of the mind during certain states of slumber,—a rapidity which would seem incredible were it not attested by the most indisputable evidence. De Quincy declares that he sometimes seemed to live 70 or 100 years in a single night. A person who was suddenly aroused from sleep by a few drops of water sprinkled in his face, dreamed of the events of an entire life, in which happiness and sorrow were mingled, and which terminated finally in an altercation upon the border of a lake, into which his exasperated companion, after a considerable struggle, succeeded in plunging him. The whole dream could have lasted but a few seconds. Dr. Abercrombie tells of a case, which is often quoted, of a man who dreamed that he had enlisted as a soldier, joined his regiment, was apprehended, carried back, tried, condemned to be shot, and at last was led out to execution. After the usual preparations a gun was fired, and he awoke with the report to find out that the cause of his disturbance was a noise in an adjoining room. In a brief space, less than it takes to describe it, this long series of events had passed through the mind. Writers on mental phenomena give many interesting instances of the same kind. There is a logical power of the mind sometimes manifested in dreams and a kind of prophetic sight, has at times momently characterized it. Condorcet, for instance, found in sleep the steps of a difficult calculation that he could not achieve during the day. I have heard persons relate similar facts. The wife of Julius Cæsar the night before his assassination dreamed that he fell bleeding across her knees. A similar condition of the mind is noticed in the case of some while falling from high altitudes, or while passing through all the sensations of drowning. The amazing swiftness of the mental operations, under suitable influences, suggests experiences of the soul in another state of being, of the most impressive character. As we think of its career with all its faculties unembarrassed, acting with their fullest capacity, where all is favorable for their most perfect movement, we are awed at the contemplation. For what volumes of strange and rich experience may it gather into itself in brief periods. What depths of knowledge may it penetrate at a glance. What areas of royal ownership, illimitable and splendid, may it sweep over in the realization of its immortality. With what a tenacity may it hold the wonders of the universe as they unroll. How the living links of its memory may be brightened and welded, until every sweet of its long past shall pour out its nectar, and every blossom of beauty that has adorned it shall shed again and again its exquisite fragrance.

The use to be made of such singular facts is to remind us of the possible achievements of the soul, its power of communicating at a distance without the usual medium, its marvellous insight, the scope and capacity of its understanding, when a sufficient degree of perfection has been attained.

The law of our being continues, whatever we ignore or notice, whether we attach a meaning to one particular manifestation of it or to another. We may never fully know ourselves. Yet were body and soul both in absolute health, then would the operations and direction of life be right and its functions be supremely blessed.

---

A small clean potato, with the end cut off, is a very convenient medium for applying brick-dust to knives, keeping it about the right moisture, while the juice of the potato assists in removing stains from the surface. A better polish can be obtained by this method than by any other we have tried, and with less labor.

